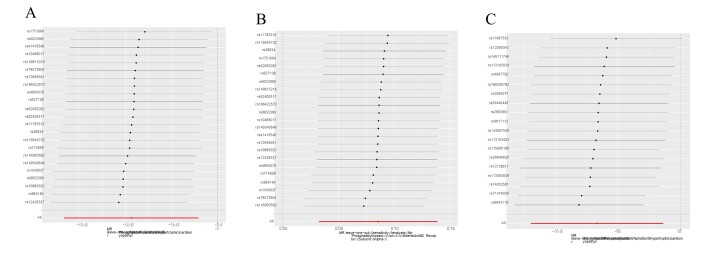# Mendelian randomization investigation: Exploring the relationship between phosphatidylinositol levels and hypertrophic cardiomyopathy risk through interleukin-20 receptor subunit alpha expression

**DOI:** 10.1097/MD.0000000000043432

**Published:** 2025-08-08

**Authors:** Luofei Huang, Jian Shi, Han Li, Quanzhi Lin

**Affiliations:** a Liuzhou Municipal Liutie Central Hospital, Liuzhou, Guangxi, China; b Department of Internal Medicine, The People’s Hospital of Laibin, Laibin, Guangxi, China; c Department of Internal Medicine, Liuzhou People’s Hospital, Liuzhou, Guangxi, China; d Department of Internal Medicine, The First Affiliated Hospital of Guangxi University of Science and Technology, Liuzhou, Guangxi, China.

**Keywords:** hypertrophic cardiomyopathy, interleukin-20 receptor subunit alpha, mediating effect, Mendelian randomization, phosphatidylinositol

## Abstract

This Mendelian randomization study investigated the causal relationship between phosphatidylinositol (PI) levels and hypertrophic cardiomyopathy (HCM) risk, while evaluating the potential mediating role of interleukin-20 receptor subunit alpha (IL-20RA). Leveraging genome-wide association study data from Finnish and European populations, our 2-sample Mendelian randomization analysis revealed a significant inverse association between PI levels and HCM risk (odds ratio [OR] = 0.686, 95% confidence interval [CI]: 0.512–0.921; *P* = .012), with no evidence of reverse causation. The analysis further demonstrated a positive correlation between PI and IL-20RA levels (OR = 1.090, 95% CI: 1.033–1.149; *P* = .002), while elevated IL-20RA showed a protective association against HCM (OR = 0.514, 95% CI: 0.302–0.873; *P* = .014). Mediation analysis indicated that approximately 15.2% of PI’s protective effect was mediated through IL-20RA pathways. These findings suggest PI may mitigate HCM risk partially through IL-20RA-mediated mechanisms, highlighting potential therapeutic targets in lipid-inflammatory pathways for HCM intervention. Further research is needed to validate these observational genetic associations.

## 1. Introduction

Hypertrophic cardiomyopathy (HCM) is a prevalent hereditary cardiovascular disorder characterized by myocardial hypertrophy, fibrosis, and diastolic dysfunction, often leading to arrhythmias, heart failure, and sudden cardiac death.^[1,2]^ Its pathogenesis involves a complex interplay of genetic mutations affecting sarcomeric proteins,^[3]^ hemodynamic stress, alterations in myocardial energetics,^[4]^ and dysregulation of signaling pathways.^[5,6]^ Current therapeutic strategies primarily focus on symptom management and risk reduction, prompting the exploration of innovative approaches targeting underlying disease mechanisms.^[7]^

Lipids are essential in cardiovascular health and disease, with distinct lipid species exerting differential effects on cardiac function and remodeling. Various lipid classes, including phospholipids, triglycerides, cholesterol, and sphingolipids, have been implicated in the pathophysiology of HCM. Inflammation is crucial in the pathogenesis of HCM, contributing to myocardial fibrosis, hypertrophy, and contractile dysfunction.^[8,9]^ Diverse lipid species can modulate inflammatory responses through multifaceted mechanisms, thereby influencing HCM progression.

Mendelian randomization (MR) analysis provides a robust framework for assessing causal relationships between modifiable exposures and disease outcomes using genetic variants as instrumental variables (IVs).^[10]^ In HCM,^[11]^ MR analysis offers several advantages, including unbiased estimation of causal effects, control for confounding factors, and temporal precedence of exposure over outcome.^[12]^ Using genetic proxies associated with lipid metabolism and inflammatory markers, MR analysis can elucidate the causal impact of lipid species on HCM risk and progression. Integrating genetic data from large-scale genome-wide association studies (GWAS) with clinical outcome data enables rigorous causal inference, guiding the development of targeted interventions and personalized treatment strategies for individuals with HCM.

## 2. Methodology and experimental protocols

### 2.1. Investigation and experimental framework

This study employed a 2-sample MR analysis to investigate the causal relationship between phosphatidylinositol (PI) levels and HCM. Our analysis focused on delineating the mediating role of interleukin-20 receptor subunit alpha (IL-20RA) levels in this association. This approach allowed us to assess the potential influence of this metabolite in the observed relationship, providing insights into the interplay of genetic and molecular factors in HCM development. Notably, MR causal inference adheres to 3 fundamental assumptions. First, a robust association exists between genetic variation and exposure variables. Second, potential confounders should not influence the relationship between genetic variation and outcomes.^[13]^ Finally, the exposure (various lipids) must serve as the sole mediator through which genetic variation influences the outcome, excluding any pleiotropic effects. This rigorous methodology strengthens the validity of our causal inferences, elucidating the role of different lipids in HCM pathogenesis based on sources of exposure and outcome data.^[14]^

### 2.2. Origins of exposure and outcome data

In the exposure data, we integrated a comprehensive array encompassing 179 lipid species. Using data from a wide-ranging series of GWAS involving 7174 Finnish individuals, we conducted univariate and multivariate genome-wide analyses of the 179 lipid species. These 179 lipids fall into 13 categories, spanning 4 major lipid classes: glycerides, glycerophospholipids, sphingolipids, and sterols.^[15]^ To analyze intermediary inflammatory factors, we conducted a genome-wide study of protein quantitative trait loci using data from 14,824 participants across multiple GWAS, where 91 inflammation-related proteins were measured using the Olink Target platform.^[16]^ Outcome measurements were derived from Finnish databases, comprising 376,233 cases of HCM and 1044 controls. This encompassed 3377,277 participants of European ancestry (Ncase = 376,233 and Ncontrol = 11,044).

### 2.3. Selection of IV

We integrated single-nucleotide polymorphisms (SNPs) demonstrating genome-wide significance (*P* < 5 × 10^–5^) and assured IV independence by employing the linkage disequilibrium reference panel from the 1000 Genomes Project. Specifically, we selected variants with *R*^2^ < 0.001 within a distance of 100,000 kb.^[17]^ To conduct MR analysis, we set a threshold of F-statistic > 10 to identify and exclude weak IVs associated with each SNP.^[18]^ We utilized multiple methodologies, including MR Egger,^[19]^ weighted median,^[20]^ inverse-variance weighted (IVW),^[21]^ simple mode, and weighted mode, to identify SNPs with consistent odds ratio (OR) values. The findings were meticulously examined based on OR values, along with tests for pleiotropy and heterogeneity.

### 2.4. Statistical analysis

The MR analysis was conducted using the MR software package (MRC Integrative Epidemiology Unit at the University of Bristol, Bristol, UK). The IVW method was employed to estimate the impact of exposure variables on outcomes based on the assumption of MR validity. Cochran *Q* test was utilized to assess residual heterogeneity in the IVW model (*P* < .05). Data visualization used scatter, funnel, and forest plots for comprehensive analysis. Additionally, scatter plots were employed to demonstrate the resilience of the results to potential outliers. Funnel plots were utilized to confirm the robustness of associations and validate the absence of heterogeneity. Finally, forest plots provided a visual representation of the interaction between IVs (SNPs) and study outcomes.

### 2.5. Feasibility assessment of the analysis

**Figure 1.**
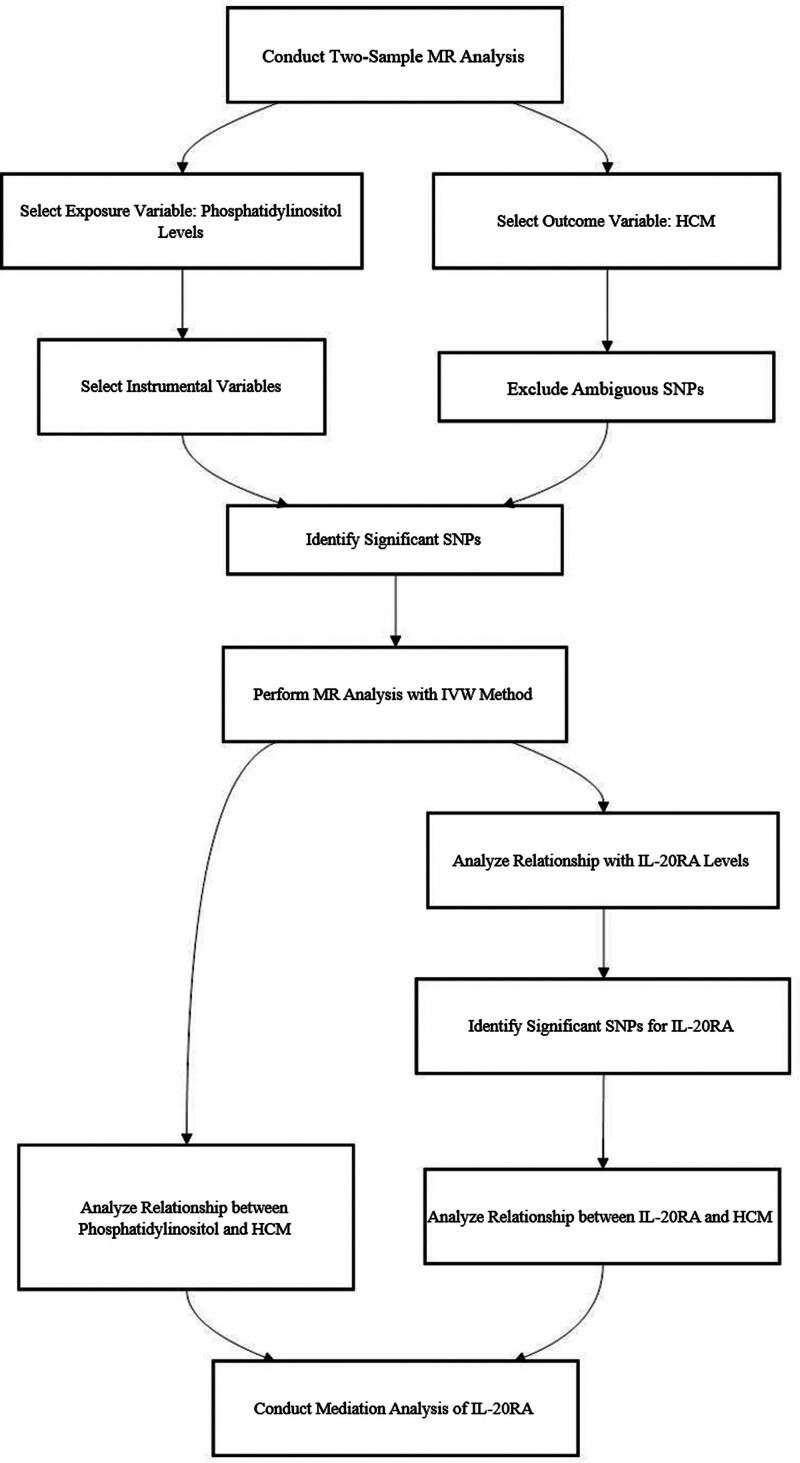
Schematic diagram of the study’s workflow. HCM = hypertrophic cardiomyopathy, IL-20RA = interleukin-20 receptor subunit alpha, MR = Mendelian randomization.

Figure 1 illustrates the framework of our methodology. We conducted bidirectional MR analyses using 2 distinct datasets to ascertain the causal relationship between PI levels and HCM. This analysis aimed to elucidate the overall impact of this association. Subsequently, mediation analysis was performed within the 2-stage MR paradigm to explore the potential mediating role of IL-20RA levels in the causal pathway linking PI levels to HCM outcomes. Our approach enables the decomposition of the composite effect into indirect (mediated) and direct (nonmediated) effects, paralleling the methods outlined in prior literature.^[22]^ Specifically, the cumulative effect of PI levels on HCM is partitioned into the direct effect of PI levels on HCM and the indirect effect of PI levels mediated through intermediate factors. The mediation ratio, calculated as the ratio of the indirect effect to the total effect, is a key aspect of our analysis, providing a comprehensive assessment of causal pathways.

## 3. Results

### 3.1. Correlation between PI levels and HCM

**Figure 2.**
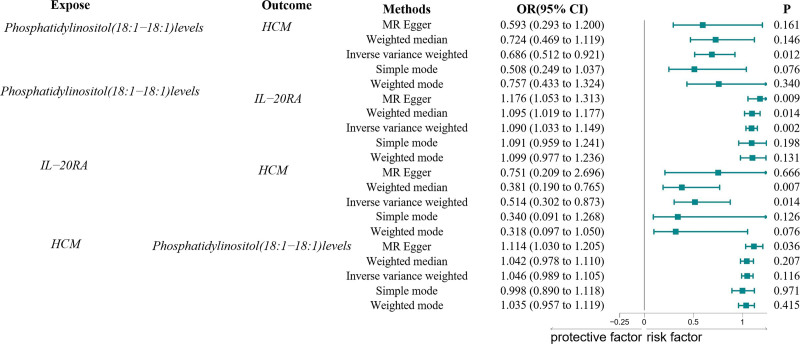
A forest plot depicting the causal associations between IL-20RA levels, PI levels, and HCM. HCM = hypertrophic cardiomyopathy, IL-20RA = interleukin-20 receptor subunit alpha, PI = phosphatidylinositol.

To investigate the causal relationship between PI levels and HCM, we conducted a 2-sample MR analysis using the IVW method. Following the selection and harmonization of IVs, all SNPs exhibited an F-statistic exceeding 10, indicating their robust instrument status. Subsequently, PI levels positively associated with HCM were chosen as the exposure variable. After excluding palindromic and ambiguous SNPs, SNPs lacking instruments, and SNPs with incorrect causal directions, 23 SNPs were identified for PI levels (Table S1, Supplemental Digital Content, https://links.lww.com/MD/P486). The OR for the IVW analysis was 0.686 (95% confidence interval [CI]: 0.512–0.921, *P* = .012). However, the MR analysis results indicated no evidence of reverse causality between genetically predicted PI levels and HCM, suggesting no causal relationship between genetically predicted PI levels and HCM. The OR was 1.046 (95% CI: 0.989–1.105; *P* = .116), calculated using the IVW method. Figure 2 illustrates these findings.

### 3.2. Correlation between PI levels and IL-20RA levels

After excluding palindromic and ambiguous SNPs, SNPs lacking proxies, and SNPs with incorrect causal directions, 23 genome-wide significant SNPs were extracted as IVs, as detailed in Table S2, Supplemental Digital Content, https://links.lww.com/MD/P486. Using the IVW method, results revealed a positive correlation between genetically predicted PI and IL-20RA levels (IVW method, OR = 1.090; 95% CI: 1.033–1.149; *P* = .002). These findings are depicted in Figure 2.

### 3.3. Correlation between IL-20RA levels and HCM

A total of 19 genome-wide significant SNPs were extracted as IVs (Table S3, Supplemental Digital Content, https://links.lww.com/MD/P486). As illustrated in Figure 2, the predicted correlation between IL-20RA levels and HCM demonstrated a statistically significant negative association (OR = 0.514, 95% CI: 0.302–0.873; *P* = .014), as determined using the IVW method. Notably, the estimated direction aligns with the IVW, MR–Egger, and weighted median methods.

### 3.4. The proportion of IL-20RA levels mediated associations between PI levels and HCM

We investigated the mediating effect of IL-20RA levels in the HCM pathway, utilizing PI levels for analysis. Our findings demonstrate an inverse correlation between increased PI levels and reduced HCM risk, while increased IL-20RA levels are associated with reduced HCM risk. Our study indicated that increased IL-20RA levels lead to an increase in PI levels, which is associated with a 15.2% decrease in HCM risk (mediation ratio: 15.2%; 95% CI = –0.78–1.09) (Table 1).

**Table 1:** Mediation ratio for the association between PI levels, IL-20RA levels, and HCM risk.

Blood lipid	Inflammatory	Outcome	Mediated effect	Mediated proportion	*P* value
Phosphatidylinositol	IL-20RA	HCM	–0.057	15.2% (–0.787, 1.090)	.751

HCM = hypertrophic cardiomyopathy, IL-20RA = interleukin-20 receptor subunit alpha, PI = phosphatidylinositol.

### 3.5. Analyzing sensitivity and pleiotropy

This study employed sensitivity analysis to examine and adjust for potential pleiotropic effects in causal estimation. Initially, we used Cochran *Q* test to assess heterogeneity among the selected SNPs, finding no significant heterogeneity within the causal relationships (Tables S4 and S5, Supplemental Digital Content, https://links.lww.com/MD/P486). Additionally, we performed a leave-one-out analysis, systematically removing each SNP and conducting an MR analysis on the remaining SNPs. The consistent results observed after each SNP’s removal further reinforced the robustness of our findings, affirming the collective contribution of all SNPs and supporting the validity of the causal relationship (Figure S1, Supplemental Digital Content, https://links.lww.com/MD/P485). This rigorous validation approach enhanced the reliability of our study, ensuring that the causal inferences were not distorted by potential pleiotropic biases.

## 4. Discussion

This study indicated an inverse relationship between PI levels and HCM risk, suggesting that elevated PI levels may have a protective effect against HCM onset of HCM. Reverse causality analysis further substantiated this hypothesis, revealing no significant impact of increased HCM risk on PI levels, thereby supporting the potential protective role of PI. Additionally, a positive correlation was observed between PI and IL-20RA levels, as well as a negative correlation between IL-20RA levels and HCM risk. These findings suggest that elevated PI levels may indirectly reduce HCM risk by enhancing IL-20RA levels, with IL-20RA acting as a mediating factor in this pathway. The proportion of the mediation effect was estimated at 15.2% (95% CI = –0.78 to 1.09). Overall, this study uncovers a complex regulatory relationship between PI and HCM risk, highlighting the mediating role of IL-20RA within this regulatory mechanism.

Furthermore, PI, a critical phospholipid, serves as a precursor for phosphoinositides and diacylglycerol, which are essential second messengers in cellular proliferation, differentiation, and apoptosis signaling pathways. Additionally, PI and its phosphorylated derivatives, especially those involved in the Phosphoinositide 3-kinase (PI3K) pathway, are significant in the onset and progression of HCM. The PI3K/protein kinase B (Akt) signaling pathway is a central mediator in cellular growth, survival, and metabolism, intricately involved in both cardiac physiology and pathology, including HCM.^[23,24]^ The PI–HCM interaction often involves the activation of the PI3K/Akt pathway. This activation can be triggered by various stimuli, including growth factors, mechanical stress, and neurohormonal activation, commonly observed in the cardiac hypertrophic response. Upon activation, PI3K phosphorylates PI 4,5-bisphosphate to generate PI 3,4,5-trisphosphate, a second messenger crucial in activating downstream signaling molecules, including Akt. Once activated, Akt phosphorylates downstream targets that promote cell survival, growth, and proliferation. These effects contribute to the hypertrophic growth of cardiomyocytes, a hallmark of HCM.^[25]^ Akt-mediated phosphorylation also inhibits apoptosis (programmed cell death) and promotes cell survival under conditions that might otherwise lead to cell death. Akt activation leads to the stimulation of the mammalian target of the rapamycin (mTOR) pathway, further promoting protein synthesis and cellular growth. The mTOR pathway is known to contribute to the enlargement of cardiomyocytes (cellular hypertrophy), a key factor in HCM development.^[26]^ Besides, PI influences calcium Ion (Ca^2+^) handling in cardiomyocytes, which is crucial for cardiac contractility. Changes in Ca^2+^ signaling and homeostasis promote the pathophysiology of HCM, affecting the heart’s contractile function and propensity for arrhythmias.^[27]^ Furthermore, the PI3K/Akt pathway affects the behavior of fibroblasts and the production of extracellular matrix proteins, leading to fibrosis, which is frequently observed in HCM. For instance, in a pressure-overload-induced mouse model of HCM, activation of the PI3K/Akt pathway significantly promotes myocardial cell hypertrophy and fibrosis. Conversely, administration of a PI3K inhibitor alleviates the degree of myocardial hypertrophy by suppressing the mTOR signaling pathway.^[26]^

Fibrosis causes cardiac stiffness, impairing filling and exacerbating functional limitations in HCM.^[28]^ This analysis highlights the multifaceted role of PI and its derivatives in HCM, suggesting that targeting the PI3K/Akt pathway could offer therapeutic potential for managing the condition. Future research should continue to elucidate the complex molecular mechanisms underlying HCM, paving the way for novel therapeutic interventions. Our study provides evidence that alterations in PI levels may impact IL-20RA expression, thereby influencing HCM development or progression. The IL-20RA is intricately involved in HCM pathogenesis through its pivotal role in modulating inflammatory responses, fibrosis, and myocardial function. The IL-20RA-mediated signaling pathways contribute to cardiac hypertrophy by activating pro-inflammatory cytokines, which induce hypertrophic pathways in cardiomyocytes and the myocardial microenvironment. This leads to increased cardiomyocyte size and the re-expression of fetal genes, a hallmark of cardiac hypertrophy.^[29]^ Moreover, IL-20RA influences fibroblast activity and extracellular matrix production, promoting fibrosis that contributes to the structural and functional remodeling characteristic of HCM.^[30]^ Its interaction with ligands affects cardiac contractility and progression to heart failure, a common complication of HCM, by altering gene expression related to Ca^2+^ handling and contractile function.^[28]^ Furthermore, the role of IL-20RA is amplified through cross-talk with other critical signaling pathways, such as PI3K/Akt and mitogen-activated protein kinase, which regulate cell survival, proliferation, and differentiation, thereby exacerbating the pathological processes underlying myocardial hypertrophy and fibrosis.^[31]^ The multifaceted impact of IL-20RA on HCM underscores the potential for targeting IL-20RA signaling as a therapeutic strategy to mitigate inflammation-induced myocardial damage and improve cardiac function and outcomes in patients with HCM.

Overall, our study offers compelling evidence of the intricate relationships between lipid metabolism, inflammation, and HCM. It emphasizes the importance of a holistic approach to understanding cardiovascular diseases, incorporating genetic, molecular, and environmental perspectives. Future research should focus on translating these findings into clinical applications, exploring novel therapeutic targets, and optimizing treatment strategies to improve outcomes for individuals with HCM. Clinical trials of “multi-target combined intervention” can be designed based on the pathways suggested by MR analysis. For example, combined administration of PI3K inhibitors and IL-20RA antagonists can be used to evaluate the efficacy of synergistically improving myocardial hypertrophy and fibrosis. While our study has made significant strides in elucidating the complex interactions between PI metabolism, IL-20RA expression, and HCM pathogenesis, several limitations remain. First, the inherent nature of MR studies, including ours, may introduce biases related to genetic pleiotropy, where genetic variants used as IVs influence the outcome through pathways other than those being studied. Although we employed multiple methodologies to mitigate this, the possibility of residual pleiotropic effects cannot be entirely excluded. Second, our analysis relies on observational data, which, despite rigorous statistical adjustments, cannot fully establish causality. The observed associations might be influenced by unmeasured confounding factors that were not considered in our model. This limitation underscores the need for experimental validation of our findings in vitro and in vivo to confirm the causal relationships suggested by our MR analysis. Third, our study focused predominantly on the PI and IL-20RA pathways without exploring the full spectrum of molecular interactions and signaling pathways involved in HCM. The complexity of HCM pathogenesis suggests that multiple pathways and genetic factors are likely involved, necessitating a broader investigation beyond the scope of the current analysis. Furthermore, genetic background and environmental factors may play distinct roles in HCM pathogenesis across different ethnic groups. Certain genetic variants are prevalent in specific populations, potentially influencing susceptibility to myocardial hypertrophy. To ensure broad applicability, future studies should include a more racially and ethnically varied sample base. In addition, to better understand the multidimensional effects of HCM, it is advisable to incorporate patient samples from diverse geographic regions and utilize a broader array of genomics, transcriptomics, and metabolomics data. This inclusive research approach will help identify ethnicity-specific differences and reveal core pathogenic mechanisms relevant across populations, advancing the development of personalized therapies.

## 5. Conclusion

Our MR study illuminated the complex interplay between PI metabolism, IL-20RA expression, and HCM pathogenesis, highlighting the significant roles of lipid metabolism and inflammatory pathways in the disease’s development and progression. Future studies should validate our findings through experimental models and explore novel therapeutic targets with an interdisciplinary approach to translate these insights into clinical practice, ultimately enhancing outcomes for individuals with HCM.

## Acknowledgments

We extend our heartfelt gratitude for the wealth of resources provided by the public database, which has offered invaluable support to our work and research.

## Author contributions

**Formal analysis:** Quanzhi Lin.

**Funding acquisition:** Quanzhi Lin.

**Methodology:** Jian Shi.

**Resources:** Han Li.

**Software:** Han Li.

**Validation:** Quanzhi Lin.

**Visualization:** Luofei Huang.

**Writing – original draft:** Luofei Huang.

**Writing – review & editing:** Jian Shi, Han Li, Quanzhi Lin.

## Supplementary Material



**Figure:**